# Electrospun micro- and nanofiber tubes for functional nervous regeneration in sciatic nerve transections

**DOI:** 10.1186/1472-6750-8-39

**Published:** 2008-04-11

**Authors:** Silvia Panseri, Carla Cunha, Joseph Lowery, Ubaldo Del Carro, Francesca Taraballi, Stefano Amadio, Angelo Vescovi, Fabrizio Gelain

**Affiliations:** 1 Bioscience and Biotechnology Department, University of Milan-Bicocca, Piazza della Scienza 2, Milan, Italy; 2 Department of Chemical Engineering and Institute for Soldier Nanotechnologies, Massachusetts Institute of Technology, 500 Technology Sq, Cambridge, USA; 3 Laboratory of Neurophysiology, IRCCS San Raffaele, Via Olgettina 58, Milan, Italy

## Abstract

**Background:**

Although many nerve prostheses have been proposed in recent years, in the case of consistent loss of nervous tissue peripheral nerve injury is still a traumatic pathology that may impair patient's movements by interrupting his motor-sensory pathways. In the last few decades tissue engineering has opened the door to new approaches;: however most of them make use of rigid channel guides that may cause cell loss due to the lack of physiological local stresses exerted over the nervous tissue during patient's movement. Electrospinning technique makes it possible to spin microfiber and nanofiber flexible tubular scaffolds composed of a number of natural and synthetic components, showing high porosity and remarkable surface/volume ratio.

**Results:**

In this study we used electrospun tubes made of biodegradable polymers (a blend of PLGA/PCL) to regenerate a 10-mm nerve gap in a rat sciatic nerve *in vivo*. Experimental groups comprise lesioned animals (control group) and lesioned animals subjected to guide conduits implantated at the severed nerve stumps, where the tubular scaffolds are filled with saline solution. Four months after surgery, sciatic nerves failed to reconnect the two stumps of transected nerves in the control animal group. In most of the treated animals the electrospun tubes induced nervous regeneration and functional reconnection of the two severed sciatic nerve tracts. Myelination and collagen IV deposition have been detected in concurrence with regenerated fibers. No significant inflammatory response has been found. Neural tracers revealed the re-establishment of functional neuronal connections and evoked potential results showed the reinnervation of the target muscles in the majority of the treated animals.

**Conclusion:**

Corroborating previous works, this study indicates that electrospun tubes, with no additional biological coating or drug loading treatment, are promising scaffolds for functional nervous regeneration. They can be knitted in meshes and various frames depending on the cytoarchitecture of the tissue to be regenerated. The versatility of this technique gives room for further scaffold improvements, like tuning the mechanical properties of the tubular structure or providing biomimetic functionalization. Moreover, these guidance conduits can be loaded with various fillers like collagen, fibrin, or self-assembling peptide gels or loaded with neurotrophic factors and seeded with cells. Electrospun scaffolds can also be synthesized in different micro-architectures to regenerate lesions in other tissues like skin and bone.

## Background

Nerve injuries usually complicate successful rehabilitation of patients because mature neurons do not replicate. However, under the right conditions, axon extensions can regenerate over small gaps caused by injury, reconnecting with the distal stump and eventually reestablishing its function. In the case of small injuries, current treatments for severed nerves typically rely on microsuture of the nerve stumps. If substantial loss of nervous tissue occurs, clinical treatment involves donor nerves obtained from a second operative site of the patient, such as an autologous nerve graft, vein graft, or arterial graft. This method is far from being the gold standard though, because its benefits have to be counterbalanced by function loss at the donor sites, formation of potential painful neuromas, structural differences between donor and recipient grafts preventing a successful regeneration, and shortage of graft material for extensive repair [1,2].

Consequently, nerve transection is still a traumatic pathology that can impair patient's movements by interrupting their motor-sensory pathways.

With the specific aim of avoiding the afore-mentioned problems, artificial grafts (also known as nerve guide conduits) have been of great interest in recent years and various attempts have been reported in the literature [3-11]. Researchers have tested tubular nerve guides made of biomaterials like poly(phosphoester) [2,12], polyethylene [13], silicon [14,15], polytetrafluoroethylene [16], collagen [14,17-19], polyglycolide [20], collagen and poly-glycolide [21], poly(L-lactide-co-glycolide) (PLGA) [22,23], poly-L-lactic acid/caprolactone [5,24-27]. Nerve guide conduits fabricated from biodegradable polymers are preferable to non-biodegradable polymers because of the obvious advantage of eliminating a second surgery to remove the conduit. If the conduit is not removed after nerve regeneration, it leads to problems such as chronic tissue response or nerve compression [28].

The fabricated conduits reported in the literature thus far usually possess a solid rigid structure. The present work deals with the fabrication of electrospun fibrous tubular constructs to act as nerve guidance channels. It is not rigid and consequently well adaptable to the living system. Electrospinning represents an attractive approach to the fabrication of fibrous biomaterials, which can mimic the size scales of fibers composing the extracellular matrix of native tissues and organs. Hence, this method represents an attractive approach to the fabrication of fibrous biomaterials for tissue engineering purposes [29,30]. While a few approaches have been attempted with electrospun poly(DL-lactide-co-glycolide) (PLGA) nerve conduits [31,32], final results did not go beyond morphometric analysis of the regenerated fibers.

We present a study comprising composite scaffolds for nerve regeneration in lesioned rats. The supportive frame is a multi-scaled guide tube made of electrospun microfibers of PLGA and poly(ε-caprolactone) (PCL). Quantified results reveal neural reconnections of the sectioned stumps along nerve conduits four months after surgery. Myelination of the regenerated fibers has been detected. Neural tracers crossed the regenerated gaps and evoked potentials have been detected at the reinnervated target muscles.

## Results

### Scaffold characterization and gross findings

**Figure 1:**
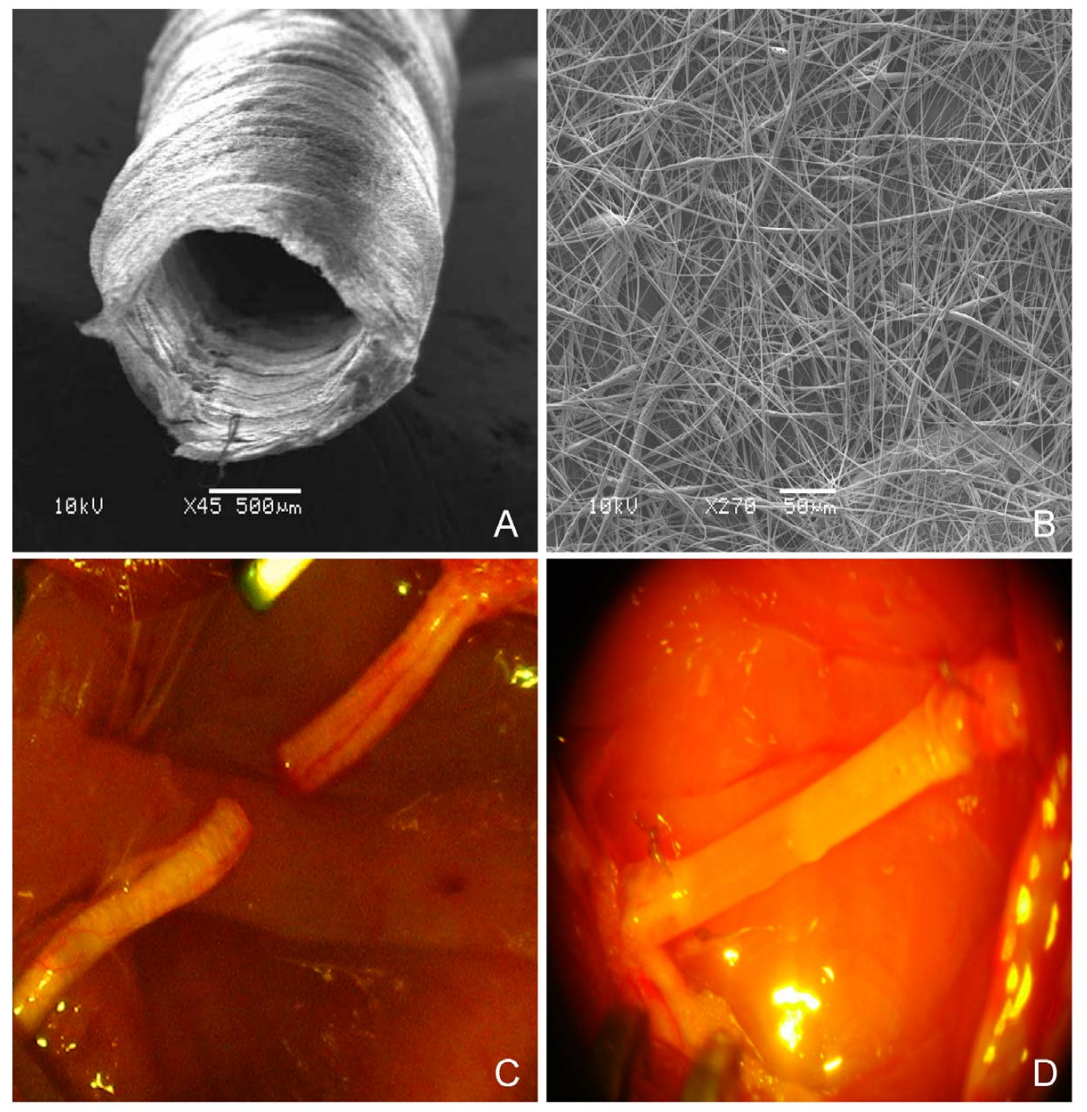
**Experimental model**. SEM images of the electrospun PLGA/PCL nerve guide conduit **(A) **and magnified details of the tube wall (**B**): microfibers and nanofibers range in diameter from approximately 280 nm to 8 μm. The non-woven fibrous microstructure is characterized by small pores (700 nm) and large pores (20 μm). **(C**) Micrograph of sham-operated rat sciatic nerve (experimental group 1). (**D**) Micrograph of prosthesis implanted, filled with saline solution and sutured to the transected nerve (experimental group 3).

A novel electrospun biodegradable micro- and nanofiber scaffold was developed by electrospinning solutions of PCL and PCL/PLGA (fig. 1A,B). Fibers ranged in diameter from approximately 280 nm to 8 μm (see methods for details). A fibrous structure was preferred over stiff continuous tubes to obtain nerve guides with high flexibility, high porosity, high surface/volume ratio favouring protein adsorption and fibrous structures that were easy to suture to the sciatic nerve stumps (fig. 1D). The fibrous micro-structure of PCL/PLGA provides mechanical stability to soft tissues, while the nanostructure adds more substrate surface for cell attachment (and therefore a higher cell density per unit of space) compared with other structures and guarantees a high-permeability of the guide walls to allow for nutrient exchange. SEM analysis of the PCL/PLGA guides showed pores with variable dimensions but uniformly distributed on the longitudinal and cross-sections with small pores (700 nm) and large pores (20 μm).

At four months after surgery the nerve tube was still present in every examined animal: neither septic collections nor tube breakages were found in the surgical field. Formation of a thin fibrous tissue capsule external to the nerve guide conduit walls was observed and macroscopically no inflammatory response was noticed in any of the treated animals, indicating good tissue response to the synthetic conduit. Tubular conduits collapsed in 16 (40%) of the treated rats due to prosthesis displacement and subsequent muscle compression exerted during the pacing of the rat. Collapsed tubes have not been considered in the following results.

In all rats of group 1 (transected sciatic nerve, fig. 1C) and group 2 (10-mm nerve gap left between the transected stumps) nervous tissue did not reconnect the two stumps of transected sciatic nerves and neural sprouting following injury did not show any significant difference between the two experimental groups. Hence group 1 and 2 are considered together and named as the control group hereafter. A spontaneous and random neural sprouting occurred from proximal stumps; however, nervous fibers targeted muscles located near the lesion site and the reconnection of the distal nerve segment was negligible. Distal nerve stumps in the control group showed macroscopic atrophy and neural degeneration (data not shown).

### Tissue analysis

**Figure 2:**
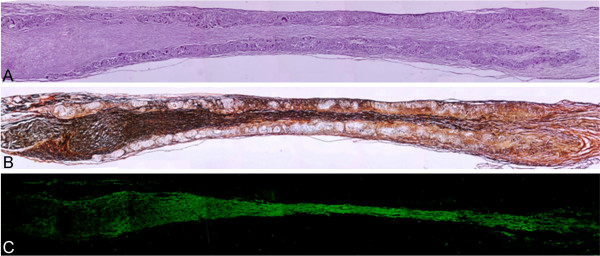
**Longitudinal sections of nerve regenerated within the implanted guide channel**. In the conduit, the regenerated nerve bridged the 10-mm gap, reconnecting the two sciatic nerve stumps.**(A) **4 months after surgery hematoxylin-eosin staining shows the presence of regenerated tissue filling the conduit lumen; decreased lumen diameter is observable at middle length of the guidance channel. Regenerated tissue positive to Bielschowsky staining **(B) **and to anti β-tubulin antibody **(C) **shows nervous projections oriented along the major axis of the prosthesis bridging the 10-mm gap between the severed sciatic nerve stumps (image sequence collected at 4× magnification).

Regenerated tissue filled the inner lumen of the nerve guidance channels in all treated rats, thus bridging the 10-mm gap between the two nerve stumps. Neurite outgrowth has been found to be mainly oriented along the longitudinal conduit axis (fig. 2A).

No significant cavities or cysts have been detected in the regenerated tissue. Most of the regenerated tissue grown inside the guide channels was positive to Bielschowsky staining (fig. 2B) and to anti β-tubulin antibody (fig. 2C). Occasionally, nervous regeneration from the proximal nerve stump was detected on the prosthesis outer edge walls concomitantly with inner lumen regeneration. In that case, Bielschowsky and β-tubulin positive fibers infiltrated and crossed the tube wall outward and vice-versa. When outer nervous regeneration was absent, tube walls were infiltrated with fibrotic tissue only.

**Figure 3:**
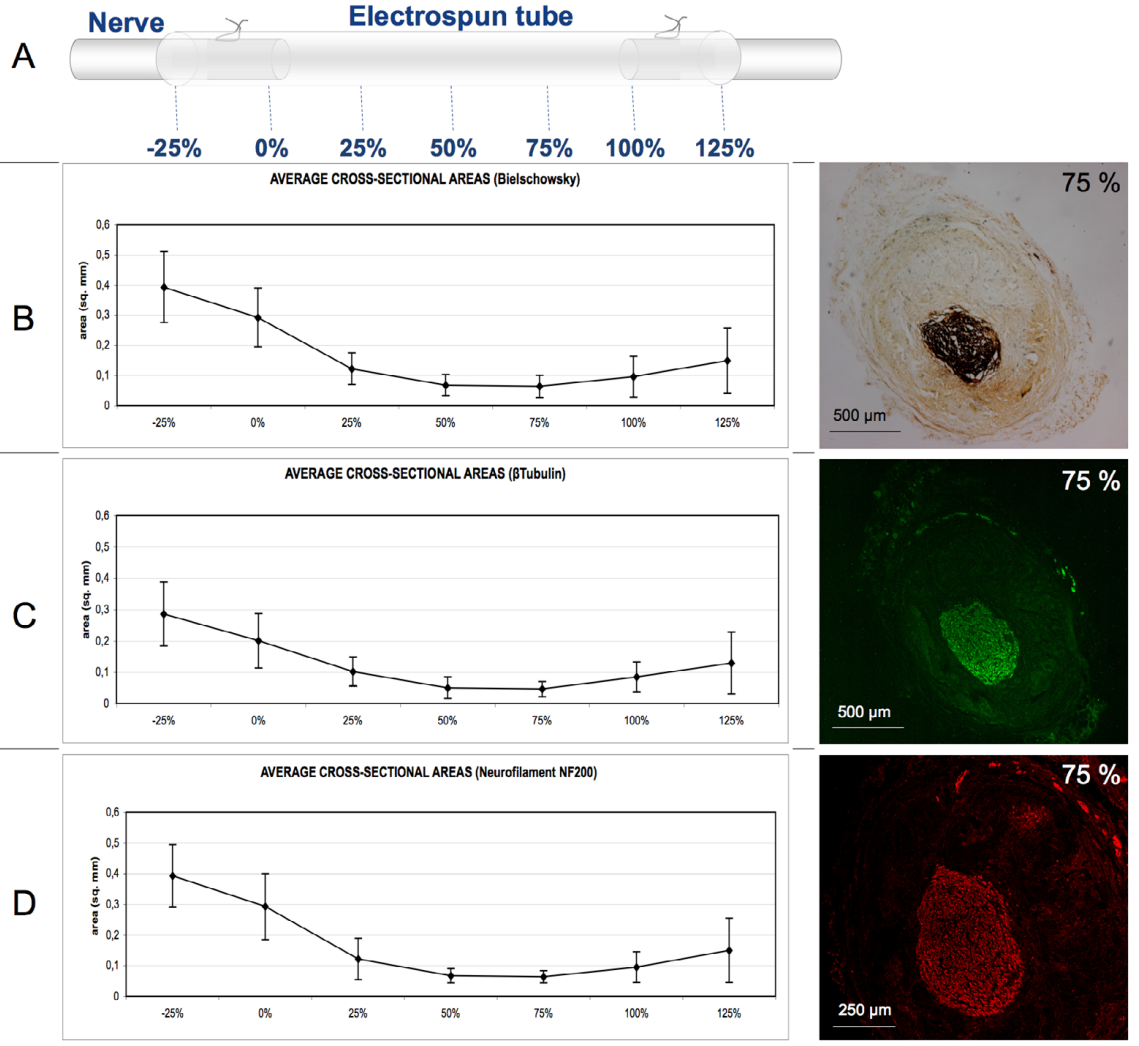
**Morphometrical analysis of nervous regenerated area**. **(A) **Scheme of the arbitrary coordinates representing the distance from the proximal side as a percentage of the total conduit length adopted to localize the estimated nervous regenerated cross-section. Cross-sectional area measurements positive to Bielschowsky reaction **(B)**, to anti β-tubulin antibody **(C) **and to anti NF200 antibody **(D) **stainings are comparable and reveal regeneration throughout the conduit lengths. Images of regenerated area positive for the aforementioned stainings detected at 75% of the conduit length are shown on the right side of each graphic. Values for the regenerated area decrease at the middle length of the conduit. Area quantification is indicated in square mm. Error bars represent standard deviation (N = 12).

In order to quantify nervous regeneration, image analysis was performed on sciatic nerve transversal sections of 12 treated rats. Acquired images were processed to quantify the nervous regenerated area at specific equally spaced distances from the proximal stumps named as percentage distances over the total gap length (fig. 3A). Measurements of the cross-sectional area positive to Bielschowsky staining, anti β-tubulin, and anti neurofilament NF200 antibody show similar values. Positive areas reveal nervous regeneration throughout the conduit length. Values show a minimum at approximately the middle conduit length (fig. 3B,C).

**Figure 4:**
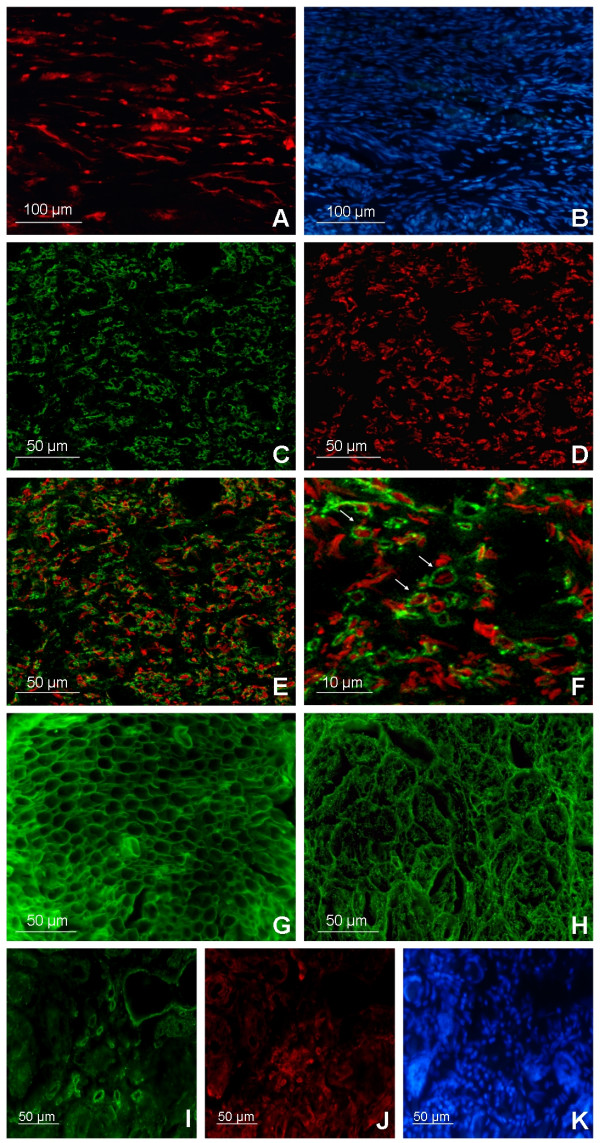
**Immunofluorescence analysis**. **(A) **Fluorescence imaging of a longitudinal section located distally to the implants shows cells positive to Fluororuby, a neural tracer injected proximally to the implanted prosthesis. Positive cells for the neural tracer in the nervous regenerated area were identified in all rats receiving the Fluororuby injection. **(B) **DAPI cell nuclei staining of the neuronal tracer positive area. Confocal cross-section image (75% of the conduit length) of myelin sheets, stained with MBP and CNPase **(C)**, wrapping regenerated axons positive to β-tubulin staining **(D)**. Myelinated axons were noticed throughout the tissue regenerated inside the conduits. **(E) **Confocal merge image shows distribution of both myelinated and unmyelinated axons in the nervous area regenerated in the conduit lumen. **(F) **High magnification confocal image, arrows indicate myelinated axons. Cross-section immunostainings for collagen IV in healthy sciatic nerves **(G) **and in regenerated tissues **(H) **show a noticeable amount of basement membrane component distributed throughout the prosthesis lumens of all the treated rats but with a remarkably different microstructure organization. Collagen IV is also detected both nearby and within the conduit inner walls **(I) **where low fibroblast density **(J) **is observable: same field DAPI cell nuclei staining **(K)**.

Fibers positive to Fluororuby, a neural tracer injected proximally to the implanted prosthesis (see methods for details), were identified in all treated rats receiving the injections both at proximal and distal stumps, concomitant to the nervous regenerated areas (fig. 4A, B). No neural tracer-positive fibers have been found distal to the injuries in control groups.

CD68 staining of the regenerated tissue within the inner lumen of the electrospun scaffolds, identifying both macrophage and pluri-nucleated foreign body giant cells [33,34] infiltration, was comparable in distribution and amount to that of healthy nerves (data not shown). On the other end, pluri-nucleated CD68 positive cells were detected within the tube walls, indicating a chronic foreign body reaction to the implanted tubular guides.

Myelinated axons were noticed throughout the tissue regenerated inside the conduits. Confocal images of the nervous area inside the conduit revealed noteworthy distribution of regenerated myelinated fascicles (MBP, CNPase, β-tubulin staining) at three-quarters of the regenerated gap lengths (fig. 4C–F), as well as at the distal nerve stumps.

Collagen IV, one of the main components of the basal lamina in nervous tissue, was found in noticeable amounts and evenly distributed throughout the implant lumens in treated animals. However, its spatial organization (fig. 4H) was not as well organized as in healthy nerves (fig. 4G). The spatial disorganization of the regenerated tissue is further highlighted by a higher density of DAPI positive cell nuclei (fig. 4B–K) with respect to the healthy nerves (data not shown): this is consistent with the loss of cytoarchitecture and proper three-dimensional cell distribution in the regenerated tissue. A low density of fibroblasts was found in the conduit lumen four months after surgery, although higher fibroblast concentrations were detected both nearby and within the conduit inner walls (fig. 4I–K).

### Behavioural test

**Figure 5:**
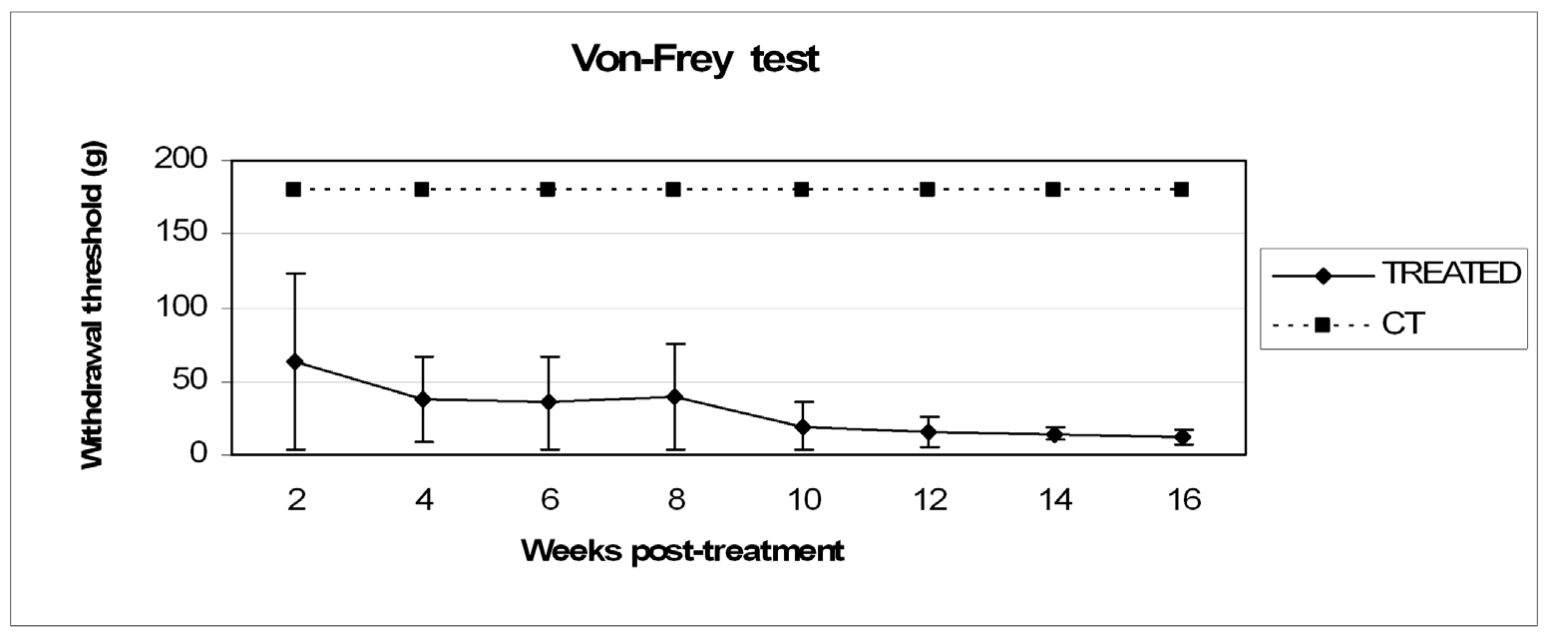
**Behavioural test results**. Animals were tested for sensory recovery using Von-Frey test every two weeks. The trend of the treated group's withdrawal threshold is shown: minimum values (12 ± 5.1 gr) were obtained at 16 weeks after surgery. The control group showed no positive response to the stimuli within the test range throughout the four months: an arbitrary value of 180 gr withdrawal threshold, corresponding to the maximum detectable threshold, has been indicated. Some animals were excluded from the mechanical stimuli test upon occurrence of spontaneous autophagy of the tested lower limb. To note, contralateral healthy nerves showed a sensitivity threshold of approximately 15 gr. Error bars represent standard deviation (N = 12).

Every two weeks treated rats were tested for their reaction to mechanical stimulation with calibrated Von-Frey hairs (see methods for details). The presented results consider a total of 12 animals since the remaining were excluded from the mechanical stimuli test upon occurrence of spontaneous autophagy of the tested lower limb. In the treated group, the withdrawal threshold, calculated using the "up and down" method, showed a decreasing trend of sensitivity threshold with a minimum at the end of the experimental time frame at 12 ± 5.1 gr (fig. 5). The control group showed no positive response to the stimuli within the test range throughout the four months, thus it has been indicated an arbitrary value of 180 gr withdrawal threshold corresponding to the maximum detectable threshold.

To note, contralateral healthy nerves showed a sensitivity threshold of approximately 15 gr.

### Neurophysiological results

**Figure 6:**
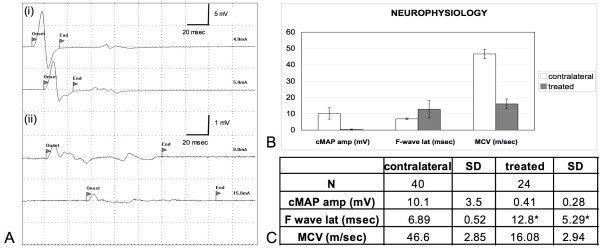
**Neurophysiological results**. No cMAP were recorded in both control group rats and in animals with collapsed prostheses. **(Ai, Aii) **The presence of the cMAP at the plantar muscles following sciatic nerve stimulation proximally to the implants was recorded in 24 of the treated rats. **(B), (C) **Mean cMAP amplitude as well as mean MCV (motor conduction velocity) are both significantly lower in the treated nerves in comparison with healthy contralateral nerves (p < 0.0001) showing a relatively early phase of nerve fibers regeneration and myelin repair after lesion. F-wave was recorded in 14 of the treated rats. (*) When detected in regenerated nerves, mean F-wave latency values are higher than in contralateral nerves.

Four months after surgery, no animal belonging to the control group showed the presence of the cMAP (compound Motor Action Potential). No cMAP was recorded in the case of collapsed prostheses, which, as stated previously, were not considered in the following results. 24 (70.6%) of the treated rats showed an initial reinnervation in plantar muscles, as demonstrated by the presence of the cMAP of the plantar muscles following sciatic nerve stimulation above the site of nerve cutting and prosthesis placement (fig. 6A). Mean cMAP amplitude as well as mean MCV (Motor Conduction Velocity) were both significantly lower in the treated nerves in comparison with healthy contralateral nerves (p < 0.0001; fig. 6B,C), showing a relatively early phase of nerve fiber regeneration and myelin repair after lesion. F-wave was recorded in 14 (41.2%) of the treated rats, with increased mean latency if compared to contralateral nerves.

## Discussion

Following nerve injury, axonal elongation may occur by spontaneous regenerative capacity of the peripheral nervous system [35]. However, random nervous sprouting fails to regenerate complete transections where extensive loss of tissue is involved. In the case of transected nerves, the regenerative process can be enhanced by suturing the two nerve stumps or, if any nerve tissue has been lost, by surgically bridging the gap with either tissue from a donor (autograft or allograft) or with a synthetic conduit. However, a major drawback of autografts is that they partially denervate the donor site to reinnervate the injury site. A tubular conduit, acting as a physical guide fabricated from degradable or non-degradable polymers for the regenerating nerve, can guide and facilitate peripheral nerve regeneration. A variety of conduits have been produced for bridging nerve gaps where both synthetic and natural materials have been used. Literature shows that materials with the highest regenerative activity are collagen and synthetic biodegradable copolymers of poly(DL-lactide-co-glycolide) and poly(ε-caprolactone) [36]. The flexible fibrous structure of the conduit we tested in this study (composed of PCL/PLGA degradable polymers) makes it easy to suture to the proximal and distal ends of the nerve stumps and to adapt it to the living systems. It has a porous structure, thus making it permeable to the entry of nutrients into the conduit lumen to promote the nerve regeneration. At the same time, the conduit has the necessary barrier to prevent the infiltration of unwanted tissues into the conduit from outside, as no ectopic tissues were seen in the cross section of the nerve regenerated in the inner lumen of the conduits. The developed conduit has no problem of tube breakage that is often encountered with other types of solid polymer conduits, and can easily be filled with physiologic solution or gel-like fillers like collagen and fibrin glue.

One advantage of electrospinning is that it does not involve heating or chemical reactions during tube synthesis. Thus, a material that is not stable to heat or chemical reactions, and cannot be processed by other methods, can be processed by electrospinning into microfibrous or nanofibrous form.

Thin fibrous tissue capsule formation around the surface of the conduits, no implant adhesions and negligible inflammatory response evinced a satisfactory biocompatibility of the guidance channels.

Four months after implantation we quantified important nervous regeneration throughout the conduits. Regenerated nervous projections were mostly aligned with the longitudinal conduit axis.

It is noteworthy that the proposed channels showed a lumen shrinking near the mid-point of the 10-mm gaps: this phenomena is jointly triggered by cellular infiltration within tube walls (thickening effect) and by muscle compression exerted during the pacing (compression effect).

The inner lumens of the open conduits were filled with regenerated nervous fascicles; widespread myelination of the regenerated fibers and deposition of basement membrane component collagen IV has been detected.

Functional reconnection of the severed nerve stumps has been unequivocally demonstrated by the following results: the presence of the neural tracer Fluororuby located both within and distally to the regenerated nervous tissue and the presence of muscular action potentials at the target muscles in 24 (70.6%) of the treated rats following electrode stimulations proximally to the regenerated gaps. It should be noted that the aforementioned functional recovery is still not satisfactory because of the lower nerve conduction rate, the bigger F-peak latency and the smaller amplitude of the detected cMAP respect to the contralateral nerve. Our results let us infer a relatively early phase of nerve fiber regeneration and myelin repair following lesions: it is likely that a longer experimental frame would have displayed a more extensive re-myelination and a higher nerve conduction velocity to the distal nerve.

The sensory recovery outcome obtained with the Von-Frey test corroborates the results obtained with neural tracers and cAMP detection tests. However, sensory recovery will probably need a more deep investigation especially within the first weeks after treatment because of the intrinsic variability of the test, which has previously been demonstrated [37] and the reduced number of animals analyzed, as a consequence of autophagy in both control and treated groups. Nevertheless, the trend of the treated group approaching the contralateral healthy nerves threshold is a good evidence of reinnervation.

In addition, our work, making use of a standard rat sciatic nerve transection model and widely adopted evaluation techniques, can be compared with results published by other groups testing nervous guides like silicon tubes or other biomaterials and fillers, by applying the widely accepted normalization theory developed by Yannas and co-workers [38,39]. Indeed, in the case of silicon guides the percent of nerves fitted with a nerve guide that are bridged by myelinated axons decreases below 100% at approximately 7 mm and drops below 50% (critical axon elongation) at 9.7 ± 1.8 mm. This same parameter is unquestionably higher with our electrospun nerve guides because in all (100%) open tubes we found nervous regeneration and myelinated fibers reconnecting the 10-mm nerve gaps. Thus a higher regenerative potential of our scaffolds compared to silicon tubes can be postulated.

While the multi-scaled structure of these nerve guides proved itself to be an useful improvement which makes joint use of the advantages arising from both electrospun microfiber and nanofiber tubular scaffolds, further enhancements to the proposed scaffold can be adopted in the near future by modifying the electrospinning proposed methodology in order to obtain nanofibers aligned along the longitudinal axis of the nerve guides while adopting a randomly oriented or, even better, a microbraided [32] micro structure, necessary to preserve the scaffolds mechanical properties. Indeed other works demonstrated that aligned electrospun fibers promote human Schwann cell maturation [40] and that nervous regeneration could be significantly enhanced with micropatterned conduits [41] and scaffolds comprising oriented mats [42].

Moreover, the efficacy of our nerve guides can be improved by means of polymer functionalizations [43], controlled release of growth factors, such as nerve growth factor (NGF), and/or other biological cues, such as Schwann cells or Neural Stem Cells that can be introduced along the graft to promote and guide regeneration of peripheral axons in rats [23,44-47].

## Conclusion

By quantifying nervous tissue regeneration (morphometric analyses) and animal functional recovery (neural tracers and evoked action potentials), our work has proved that multi-scaled electrospun nerve conduits are promising bioabsorbable scaffolds for stimulating and guiding peripheral nerve functional regeneration in rat models of sciatic nerve transection. Our detailed analysis of various aspects of nerve regeneration shows how microfibrous and nanofibrous prosthesis do not produce mechanical stress-related nervous degenerations and, on the other hand, favour a functional and effective nervous regeneration that could be further ameliorated via complementary strategies like hydrogels for drug delivery [48], electrical stimulation [49] and techniques adopted in clinics, such as physiotherapy [50].

## Methods

### Biopolymer electrospinning and tube synthesis

Poly(DL-lactide-co-glycolide) (PLGA, 75:25, MW 66,000–107,000) and poly(ε-caprolactone) (PCL, MW 80,000) were purchased from Sigma-Aldrich, St. Louis, Missouri. Chloroform and methanol solvents were purchased from Mallinkrodt and Sigma-Aldrich, respectively. A solution of 5.5% (W/W) PCL and 4% (W/W) PLGA, was mixed in 3:1 chloroform:methanol. A solution of 15% PCL in chloroform was also created.

The electrospinning apparatus was designed with a Gamma High Voltage Research HV power supply linked to a 16 cm-wide flat plate. A Harvard Apparatus PHD 2000 infusion syringe pump dispersed solution at a rate of 0.05 mL/min through a 35 cm Teflon tube connected to a metal needle (inner diameter: 1.06 mm) protruding through the flat plate. Fiber lumen mats were collected on a round, flat target coated with nonstick Reynolds aluminum foil. The distance between the charged plate and grounded target was 32 cm. Current was measured using a Fluke 189 True RMS Multimeter in series with a 1 Megohm resistor. Voltage was set at 34 kV for the 15% PCL solution and 25 kV for the PCL/PLGA solution [51,52].

Pure PCL fibers ranged in diameter from 2.5 μm to approximately 8.0 μm, with an average of 7.48 ± 2.02 nm. The surface of these fibers was found to be quite rough, with grooves approximately 200 nm wide running parallel to the fiber axis. PCL/PLGA fibers possessed a diameter of 279 ± 87 nm, with fibers spanning 140 nm to 500 nm.

To create the sciatic nerve tube implants, micro- and nanofibers were deposited on a 16-gauge copper wire (diameter: 1.29 mm) held near the grounded target. The wire was grounded and rotated to assure even coating. A base of larger PCL fibers was deposited for 60 seconds followed by a 120 second coating of smaller PCL/PLGA fibers (total wall thickness: approximately 155 μm). The coating of larger PCL fibers grants mechanical stability and elasticity while the smaller PCL/PLGA fibers form a tight-knit outer mesh. Samples were annealed for 24 hours at 55°C under vacuum to remove any possible residual solvent and further crystallize PCL segments for added mechanical strength.

### Surgery and tube implantation

50 female Sprague-Dawley rats weighting 200–250 gr (Charles River Laboratories, Calco, Italy) were randomly assigned to 3 groups. In group 1 (N = 5) sciatic nerves were transected. In group 2 (N = 5) a 5–7 mm segment of the sciatic nerve was removed in order to leave a 10-mm interstump gap after natural stump retraction post neurotmesis. In group 3 (N = 40) electrospun tubes filled with saline solution were implanted following neurotmesis.

Rats were anesthetized with an intraperitoneal (i.p.) injection of ketamine (80 mg/kg) and xylazine (10 mg/kg). Under aseptic conditions, the sciatic nerve was exposed by a skin incision along the femur, followed by the separation of the biceps femoris and superficial gluteal muscles. Sciatic nerves were sharply transected at the mid-thigh level, proximal to the tibial and peroneal bifurcation.

After transection, the group 1 and group 2 injured nerves were left unrepaired. In group 3 the proximal and distal nerve stumps were pulled 1.5 mm into each opening of the PLGA/PCL tube (pre-soaked in sterile phosphate buffer saline, PBS) and sutured with 8-0 Vycril (Ethicon, Somerville, NJ): the final interstump gap (10 mm long) was filled with saline solution. In all groups, muscle wound beds were sutured with 3-0 Vicryl and the skin incision was closed with surgical staples. A few drops (10 μl) of fibrin glue (Baxter, Deerfield, IL) were added at the tube ends in order to better isolate the inner lumen from the surrounding tissues. Carprofen analgesia (5 mg/kg) was administered daily for 1 week postoperatively in all groups. Rats were checked for autophagia on a daily basis.

After 14 weeks, 4 rats of group 3 received an injection of Fluororuby (FR) [7], an anterograde neuronal tracer (Molecular Probes, Eugene). Animals were anesthetized and 3 μl of FR was intraneurally injected into the sciatic nerve proximally the implanted tubes using a Hamilton syringe with a 33-gauge needle.

### Behavioral analysis

Von-Frey tests were used to observe any sensory recovery after complete sciatic nerve transection. In all three groups, rats' sensory recovery-withdrawal was performed every two weeks. The animals were placed in a plastic cage with a metal mesh floor, free to move. After an acclimatization step of 30 minutes, a series of Von-Frey filaments, ranging from 6 to 180 gr. were used. The filaments were applied perpendicularly to the plantar surface of the hindpaw with sufficient force to bend the filament for 6 seconds. Brisk withdrawal or paw licking were considered as a positive response. In the absence of a response, the filament of next greater force was applied. In the presence of a response, the filament of next lower force was applied. The tactile stimulus producing a 50% likelihood of the positive response was calculated by using the "up and down" method, as described in detail previously [53].

### Electrophysiological methods

Rats were anesthetized with ketamine (80 mg/kg) and xylazine (10 mg/kg) and placed under a heating lamp to avoid hypothermia. They were fixed with tape on a smooth table to prevent movement artefacts due to the electrical stimulation, the lower limbs gently stretched to make it easier to measure distances between proximal and distal points of stimulation.

The sciatic nerve motor conduction velocity (MCV) was obtained by stimulating the nerve with steel monopolar needle electrodes. A pair of stimulating electrodes was inserted subcutaneously near the nerve at the ankle; a second pair of electrodes was placed at the ischiatic notch, proximally to cutaneous scar, to obtain two distinct sites of stimulation, proximal and distal, along the nerve. The muscular response to the electrical nerve stimulation, named compound motor action potential (cMAP), was recorded with a pair of recording needle electrodes; the active electrode was inserted in muscles in the middle of the paw, while the reference was placed in the skin between the first and second digit. Thus, MCV was measured dividing the distance between the two points of stimulation by the difference of proximal and distal cMAPs latencies. The amplitude of cMAP and the F wave latency by stimulation of sciatic nerve at the ankle were also recorded.

Four months after nerve cutting, we studied the treated rats (N = 34) and the control rats (N = 6). In all the animals we performed bilateral sciatic MCV. As a control of the experiment, we used the MCV values of the healthy contralateral nerves of both treated and control rats (N = 40).

### Tissue collection

16 weeks after surgery, rats were deeply anesthetized with an i.p. overdose of ketamine/xylazine. Rats were perfused intracardially with a heparinised saline solution, followed by a 4% paraformaldehyde solution. In groups 1 and 2, sciatic nerves were exposed and pictures were captured by an HP digital camera; muscle tissues infiltrated by the two stumps were harvested and collected. In group 3 the sciatic nerve and the PLGA/PLC tube were removed, postfixed for 4 hrs in 4% paraformaldehyde and cryoprotected in 30% sucrose overnight. Transversal sections (16 μm thickness) were serially collected via a freezing microtome (Microm, Walldorf) for all rats, apart from the ones subjected to the FR injection, for which longitudinal sections were collected. Slices were kept at -20°C until use.

### Histochemical and immunofluorescence analysis

For histochemical analysis, slices were stained with hematoxylin-eosin and Bielschowsky reaction, a silver staining for axons (nerve fibers are sensitized with a silver solution, the sections are treated with ammoniacal silver, and then reduced to a visible metallic silver) [54]. For immunofluorescence analysis, the following primary antibodies were used: anti-neurofilament NF200 (Sigma, Sant Louis), anti-myelin MBP (Sternberger Monoclonals Incorporated, Lutherville) and anti-CNPase (Chemicon International, Temecula), anti-rat macrophage marker CD68 (Serotec, Dusseldorf), anti-prolyl 4-hydroxylase fibroblast marker (Acris Antibodies, Hiddenhausen), anti-β-tubulin (Berkeley Antibody Company, Berkeley), anti-rat Collagen IV (Cedarlene, Hornby). Primary antibodies were then probed with the secondary antibody ALEXA 488 (Molecular Probes) or CY3 (Jackson Immuno Research, West Grove). Cell nuclei were stained with DAPI (Molecular Probes). Sections were mounted with FluorSave reagent (Calbiochem, Darmstadt) and examined by an upright Nikon200 fluorescence or confocal microscope (Zeiss). In the case of neural tracer imaging, sections were mounted with FluorSave and inspected.

Confocal and upright fluorescence microscope images (4× and 10× magnification) of the sciatic nerve transversal sections of group 3 (N = 12 rats) were acquired to quantify the regenerated nerve area. Areas were quantified at specific locations representing the distance from the proximal side as a percentage of the total length: -25% and 125% represent sections obtained outside of the conduit on the proximal and distal sides respectively [41]. Measurements of the cross-sectional area positive to Bielschowsky staining and to β-tubulin and neurofilament NF200 antigens were carried out with NIH image software ImageJ. Positive pixel area was then converted to mm^2 ^scale.

## Authors' contributions

FG, SP, ALV conceived and designed the experiments. JLL synthesized and characterized the guidance channels. SP, FG, CC, FT, SA, UDC performed the experiments. FG, SP, CC, UDC analyzed the data. SP, FG, CC, ALV, UDC, JLL contributed reagents/materials/analysis tools. SP, FG, CC, ALV, UDC, JLL wrote the paper. All authors read and approved the final manuscript.
